# Macrophage Therapy for Acute Liver Injury (MAIL): a study protocol for a phase 1 randomised, open-label, dose-escalation study to evaluate safety, tolerability and activity of allogeneic alternatively activated macrophages in patients with paracetamol-induced acute liver injury in the UK

**DOI:** 10.1136/bmjopen-2024-089417

**Published:** 2024-12-09

**Authors:** Christopher Humphries, Melisande Addison, Guruprasad Aithal, Julia Boyd, Lesley Briody, John D M Campbell, Maria Elena Candela, Ellise Clarke, James Coulson, Nicholas Downing-James, Robert John Fontana, Ailsa Geddes, Julia Grahamslaw, Alison Grant, Anna Heye, James A Hutchinson, Ashley Jones, Fiona Mitchell, Joanna Moore, Alice Riddell, Aryelly Rodriguez, Angela Thomas, Garry Tucker, Kim Walker, Christopher J Weir, Rachel Woods, Sharon Zahra, Stuart J Forbes, James W Dear

**Affiliations:** 1 Centre for Cardiovascular Science, University of Edinburgh, The Queen's Medical Research Institute, 47 Little France Crescent, Edinburgh, UK; 2 Centre for Regenerative Medicine, University of Edinburgh, 5 Little France Drive, Edinburgh, UK; 3 MAIL Trial Data Monitoring Committee, Edinburgh Clinical Trials Unit, Usher Institute, University of Edinburgh, 3 Little France Road, Edinburgh, UK; 4 NIHR Nottingham Biomedical Research Centre, Nottingham University Hospitals NHS Trust, Queen's Medical Centre, Derby Road, Nottingham, UK; 5 Edinburgh Clinical Trials Unit, Usher Institute, University of Edinburgh, 3 Little France Road, Edinburgh, UK; 6 Edinburgh Clinical Research Facility, Royal Infirmary of Edinburgh, 51 Little France Crescent, Edinburgh, UK; 7 Scottish National Blood Transfusion Service, Jack Copland Centre, 52 Research Avenue North, Edinburgh, UK; 8 Emergency Medicine Research Group Edinburgh, Royal Infirmary of Edinburgh, 51 Little France Crescent, Edinburgh, UK; 9 MAIL Trial Steering Committee, Edinburgh Clinical Trials Unit, Usher Institute, University of Edinburgh, 3 Little France Road, Edinburgh, UK; 10 Centre for Precision Cell Therapy for the Liver, Lothian Health Board, Queen's Medical Research Institute, 47 Little France Crescent, Edinburgh, UK

**Keywords:** Hepatology, Toxicology, Clinical Trial

## Abstract

**Introduction:**

Acute liver failure (ALF) has no effective treatment other than liver transplantation and is commonly caused by paracetamol overdose. New treatments are needed to treat and prevent ALF. Alternatively-activated macrophages (AAMs) can promote resolution of liver necrosis and stimulate hepatocyte proliferation. Using AAMs in unscheduled care requires the use of an allogeneic product. A clinical trial is needed to determine the safety and tolerability of allogeneic AAMs.

**Methods and analysis:**

A single-centre, open-label, dose-escalation, phase 1 randomised trial to determine whether there is dose-limiting toxicity of AAMs in patients with paracetamol-induced acute liver injury. Randomisation will occur at higher doses. Between 17 and 30 patients will receive treatment, subject to dose-limiting toxicity and an adaptive trial design which aims to reduce the risk of allocation bias through blinding and randomisation.

**Ethics and dissemination:**

The trial will be conducted according to the ethical principles of the Declaration of Helsinki 2013 and has been approved by North East—York Research Ethics Committee (reference 23/NE/0019), National Health Service Lothian Research and Development department, and the UK Medicines and Healthcare products Regulatory Agency. When the trial concludes, results will be shared by presentation and publication.

**Trial registration number:**

ISRCTN12637839.

**STRENGTHS AND LIMITATIONS OF THIS STUDY**

- This phase 1 study uses randomisation with concealed allocation to reduce the risk of allocation bias.
- The study will use a variety of biomarkers and clinical assessment to identify the safety and tolerability of allogeneic macrophages in humans with acute liver injury.
- This study has the potential to lay the foundation for a phase 2 clinical trial of an ‘off-the-shelf’ macrophage product for acute liver injury.

## Introduction

### Background

 Acute liver failure (ALF) has no effective treatment other than liver transplantation, which has associated morbidity/mortality, expense and a scarcity of donor livers.^1^ The most common cause of ALF is paracetamol (acetaminophen) overdose (POD). In the UK, around 50 000 people need emergency treatment with acetylcysteine (n-acetylcysteine, NAC), every year.^2^ The USA has around 89 000 hospital visits annually for POD, with an estimated annual treatment cost of US$1 billion.^3^ Around 10% of these patients develop biochemical evidence of acute liver injury (ALI) and around 1 in 200 develop ALI severe enough after POD to need a liver transplant.^4^ Mortality in the ALF group is around 30%–35%, with about 30% receiving a liver transplant (most of whom survive but will require lifelong ongoing treatment and often have morbidity secondary to the immunosuppression required for transplantation).^1^ Many of these patients occupy critical care beds for weeks. Aside from paracetamol, there is a wide range of other causes for ALF including non-paracetamol drug-induced liver injury and viral infection. There is no specific treatment for ALF due to non-paracetamol causes (NAC is sometimes used but has modest efficacy).^5^

In cases of POD, the metabolic pathways that eliminate the drug become saturated and a highly reactive metabolite, N-acetyl-p-benzoquinone imine (NAPQI), is made in excess by the cytochrome P450 enzyme system. NAPQI is directly toxic to hepatocytes and leads to liver necrosis.^6^ The immune system responds by a massive infiltration of circulating inflammatory monocytes into the liver.^7^ These cells create a highly toxic inflammatory environment. In humans, the degree of infiltration of monocytes, identified by concurrent monocytopenia in the circulation, reflects the degree of ALI.^8^ Over a period of 24–48 hours, liver-infiltrating monocytes differentiate to macrophages which adopt an anti-inflammatory, wound-healing phenotype (‘M2’).^7^ These macrophages initiate the healing process and facilitate the transition from the initial inflammatory phase of ALF to the resolution phase. Macrophage-mediated resolution of ALI comprises phagocytosis of necrotic hepatocytes, induction of regeneration by proliferation of hepatocyte progenitors and suppression of systemic inflammation via an anti-inflammatory cytokine effect.^9^

Currently, the only effective treatment for preventing ALI after POD is NAC. If treatment is commenced within 8 hours of overdose, then NAC is nearly 100% effective. However, effectiveness drops substantially when treatment is delayed; NAC is nearly ineffective when treatment is delayed greater than 20 hours after overdose, though delayed treatment may reduce the subsequent development of hepatic encephalopathy and mortality.^10 11^ There is a clear unmet need for new treatments to prevent and/or treat ALF.

### Rationale for study

We urgently need a treatment which prevents the progression of ALI to ALF to prevent liver transplantation or death. Halting or reversing progression to ALF would be life-saving and remove the need for immunosuppression and morbidity/mortality associated with transplantation. The proposed solution is to develop allogeneic alternatively-activated macrophages (AAMs) as a treatment.^12^ An allogeneic product is necessary for the acute setting, when autologous monocytes may have altered properties, and there is insufficient time to produce autologous cell products.^13^ This cell therapy has clear efficacy when delivered after liver injury is established in mouse models.^14^ This is because macrophage therapy reverses injury rather than preventing it. Early phase human studies of chronic liver disease using autologous macrophage therapy have provided evidence to support the safety of AAMs, but a phase 1 trial is required to establish whether an allogeneic product is feasible.^15^

The primary aim of this trial is to determine the safety and tolerability of AAMs. There are multiple causes of ALF other than paracetamol, but POD is the most common cause in the UK and patients typically have a clearly defined onset of injury and blood tests to confirm the diagnosis. Due to the distribution and mechanisms of action of AAMs, healthy volunteers are not a suitable test population. AAMs localise to injured liver tissue, where they target necrotic hepatocytes and reduce inflammatory factors. Therefore, humans with healthy livers would not provide meaningful safety or activity data.

If successful, this study will pave the way for a randomised controlled trial of the effectiveness of macrophage cell therapy in patients with paracetamol-induced ALI and facilitate the development of macrophage cell therapy for other causes of ALF. Here, we summarise the MAIL (Macrophage therapy for Acute Injury to the Liver) trial protocol in accordance with the Standard Protocol Items: Recommendations for Interventional Trials (SPIRIT) guidance for randomised trial protocols and the SPIRIT-DEFINE extension for dose-finding studies.^16^

### Objectives

The primary objective of this phase 1 trial is to determine whether there is dose-limiting toxicity (DLT) produced by allogeneic AAMs in patients with paracetamol-induced ALI.

The secondary objectives are to explore the safety, activity and immunogenicity of allogeneic AAMs in patients with paracetamol-induced ALI.

### Trial design

MAIL is a single-centre, open-label, dose-escalation, phase 1 randomised trial to determine whether there is DLT of AAMs in patients with paracetamol-induced ALI. Randomisation will occur at higher doses. Sentinel dosing of patients and dose escalation decisions will be guided by an independent Data Monitoring Committee (DMC).

**Figure 1:**
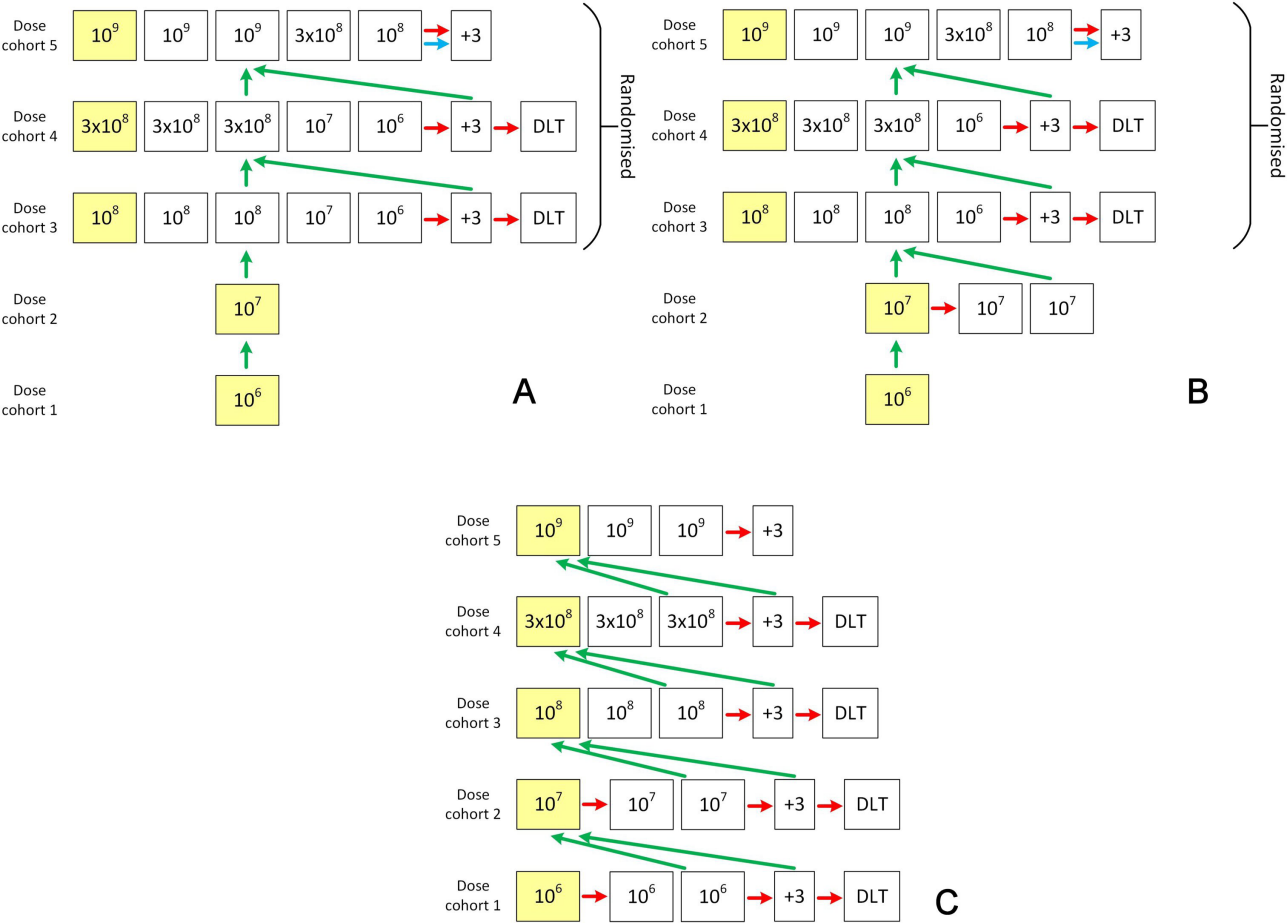
(A) Overview of MAIL trial design—scenario 1 (rapid escalation). In cohort 1, the first patient will be dosed with 106 cells. In scenario 1, there is no safety concern following DMC review and the dose is escalated to 107 (cohort 2) and then into randomised blocks in cohorts 3, 4 and 5. A green arrow represents a DMC recommendation to dose escalate. A red arrow indicates a DMC recommendation to expand at that dose. The blue arrow indicates a trial team option to expand at the highest dose. Yellow squares are sentinel dosed patients. The highest dose is up to 10^9^ cells. (B) Overview of MAIL trial design—scenario 2. In scenario 2, there is a safety signal in cohort 2 and the DMC advise two further patients are added. No further signals are observed at 107 and the 107 doses are dropped from the randomisation in cohorts 3 and 4. A green arrow represents a DMC recommendation to dose escalate. A red arrow indicates a DMC recommendation to expand at that dose. The blue arrow indicates a trial team option to expand at highest dose. Yellow squares are sentinel dosed patients. The highest dose is up to 10^9^ cells. (C) Overview of MAIL trial design—scenario 3 (3+3 escalation). In scenario 3, there is a safety concern and there is expansion of cohorts 1 and 2 into 3+3 blocks. Each square represents one patient dosed at the dose indicated, except the +3 box which indicates an option to dose three further patients at the highest dose in that cohort. A green arrow represents a DMC recommendation to dose escalate. A red arrow indicates a DMC recommendation to expand at that dose. Yellow squares are sentinel dosed patients. The highest dose is up to 10^9^ cells. DLT, dose-limiting toxicity; DMC, data monitoring committee; MAIL, Macrophage therapy for Acute Injury to the Liver.

Figure 1A describes the planned trial design. The heterogeneity in patient outcomes following the development of paracetamol-induced ALI leads to a risk of allocation bias if the next dose of cells to be given is known by the recruiting team, as less unwell patients could be preferentially enrolled at higher doses. Therefore, we will use randomisation with concealed allocation at higher dosing levels and will blind the recruiting team to prevent prediction of the next dose of AAM treatment.

Extrapolating from mouse models, we predict the clinically active dose will be between 10^8^ and 10^9^ cells. The biologically active dose in mouse models of paracetamol liver injury is 10^6^ cells (the lowest trial dose). Scaling up to humans, the predicted human equivalent dose is between 2×10^8^ (if calculated using body surface area) to 2×10^9^ (using body weight). If allometric scaling is used with liver weight and blood flow as variables the human equivalent dose of AAMs is around 10^9^ (the highest trial dose).

Within each dosing cohort, the first participant to receive the highest dose will be a sentinel dosing patient. There will be at least 1-week pause to recruitment following the conclusion of each dosing cohort. The DMC will make a decision on dose progression using data which will include all unblinded demographic, concomitant medications, infusion, physiological, safety and activity data gathered at days −1, 0, 1, 2 (if applicable) and 7 at a minimum, which will be quality-control checked before DMC review. Data from day 30 will also be included if available at the time of the DMC meeting. The sponsor, chief investigator and trial steering committee (TSC) will review the DMC recommendation and make a documented decision regarding dose progression.

For all dosing cohorts, the DMC can recommend a further three patients be dosed at the highest dose in that cohort. If one participant in a dose cohort experiences potential DLT, then the DMC can recommend treating three additional participants at the same dose. If DLT occurs in two or more patients in any size of dose cohort, then recruitment should end.

If there is a safety signal in dose cohorts 1 or 2, the DMC can recommend the addition of two extra patients to that cohort. If a further two patients are recommended by the DMC in cohort 2 (10^7^) but not in cohort 1 (10^6^) and no further signals are observed at 10^7^ (scenario 2—figure 1B), then the 10^7^ doses are dropped from the randomisation in cohorts 3 and 4 (four patients randomised: three at higher dose and one at 10^6^).

If an additional two patients are recommended by the DMC in cohort 1 (10^6^), then two patients must also be added to cohort 2 (10^7^). The trial design would thereafter effectively follow a ‘3+3’ phase 1 trial design (scenario 3—figure 1C).^17^

## Methods

### Study oversight

The MAIL trial is an investigator-led study, funded by the Medical Research Council (reference MR/T044802/1) and sponsored by ACCORD (Academic and Clinical Central Office for Research and Development for National Health Service (NHS) Lothian/University of Edinburgh, reference AC22087, ACCORD@nhslothian.scot.nhs.uk). Trial oversight is directly provided by an independent TSC and DMC, who will provide advice and review of safety. The trial has been authorised by the UK Medicines and Healthcare products Regulatory Agency (MHRA) (CTA 01384/0270/001-0001).

The composition of the TSC, steering group, regulatory and clinical trial group and DMC are provided as online supplemental file 2.

### Study setting

**Figure 2:**
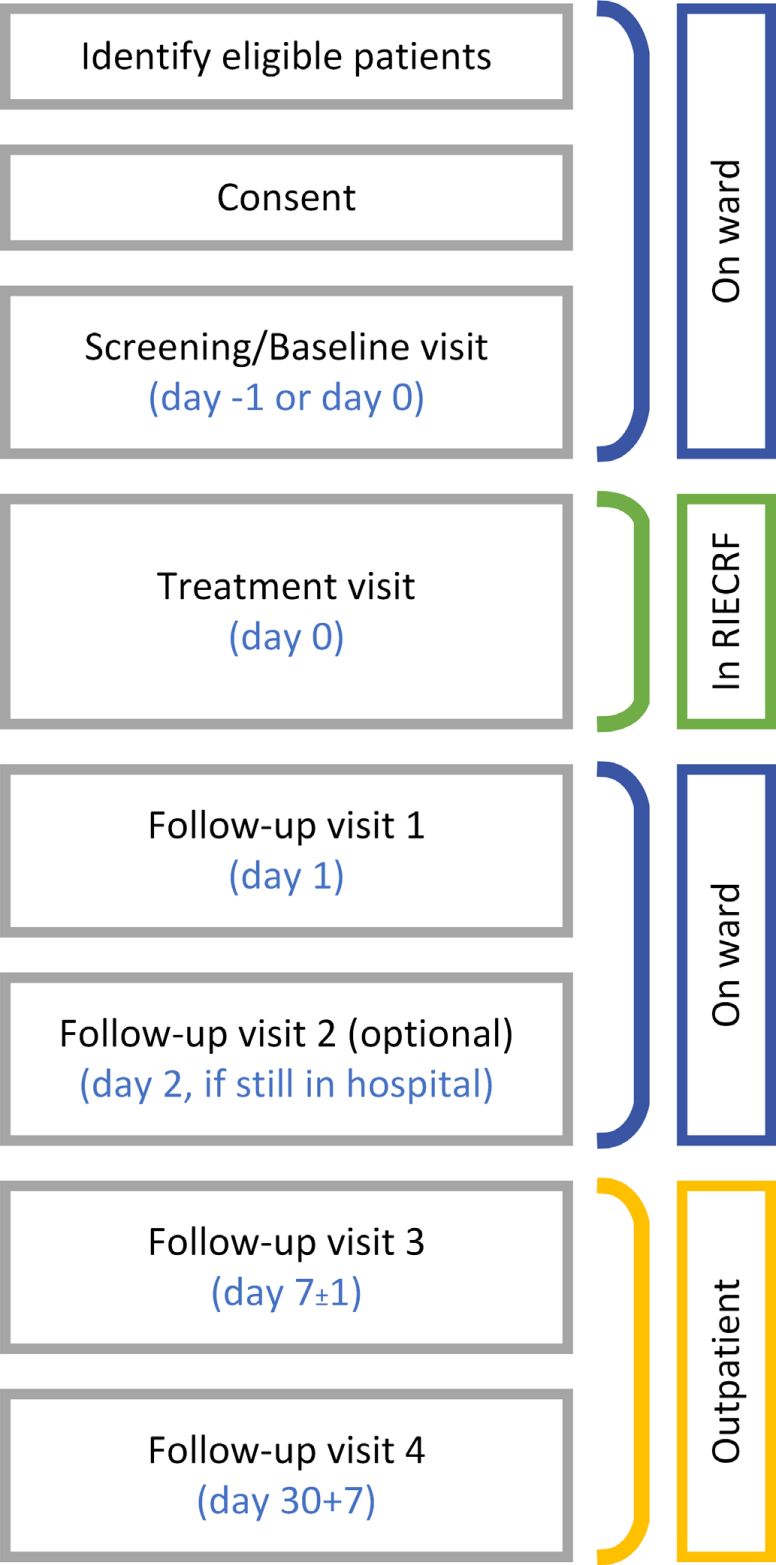
Study visits flow diagram.

This is a single-centre trial, taking place at the Royal Infirmary of Edinburgh. The recruitment commenced on 1 September 2023 and is anticipated to run for approximately 2 years. Eligible inpatients who have signed the informed consent will receive a single infusion of AAMs in the Royal Infirmary of Edinburgh Clinical Research Facility (RIECRF). Patients will remain in the RIECRF for at least 6 hours after dosing and then will return to the toxicology ward or transplant unit for continued monitoring. Patients will also receive acetylcysteine as per standard care. The period of follow-up will be 30 days. Please refer to figure 2 for a study visits flow diagram.

### Patient and public involvement

Draft versions of the participant information sheet and lay summary were reviewed by the Edinburgh Clinical Research Facility Patient Advisory Group, a well-established Patient and Public Involvement group. They will also be asked to review lay summaries of results prior to dissemination. In addition, a member of the Sheffield Emergency Care Forum has joined the TSC as an independent lay member.

### Eligibility criteria

#### Inclusion criteria

All of:

1. Serum alanine aminotransferase (ALT) activity >1000 U/L at any point between admission for the current POD and confirmation of eligibility.
2. History of POD within 5 days of ALT >1000 U/L. Overdoses of paracetamol alone and mixed overdoses are eligible.
3. Other causes of ALT increase excluded based on previous investigations and trial screening. This will be documented in the patient’s medical notes.
4. Provision of written informed consent.
5. Adult male or female (16 years old or above).*
6. Deemed safe for hospital discharge from a mental health perspective after full mental health assessment by a mental health professional. This will be documented in the patient’s medical notes.
7. Patients with childbearing potential must have a negative urine or serum pregnancy test at screening. If the patient is of childbearing potential or is a male with a female partner with childbearing potential, the patient, and their partner(s), must agree to use a highly effective method of contraception throughout the trial period and for 90 days poststudy completion.

#### Exclusion criteria

Any of:

1. Patients who do not have the capacity to consent.
2. Any situation that in the investigator’s opinion may interfere with optimal or safe study participation such as clinically relevant concurrent illness, alcohol or drug abuse, potential non-compliance, or inability to cooperate.
3. Patients with known current viral hepatitis A, B, C or E, HIV, cytomegalovirus, Epstein-Barr virus or known COVID-19 infection.
4. Patients who are pregnant, or are planning on becoming pregnant during the study, or are breast feeding and wish to continue breast feeding. Patients of childbearing potential, or male patients with a female partner of childbearing potential, who do not agree to use a highly effective method of contraception as detailed above.
5. Patients who have previously participated in this study or another ATMP.
6. Potentially life-threatening liver injury with an immediate need for transplantation as documented in the patient’s medical notes.
7. Patients who are listed for or previously received any organ transplant.
8. Patients with stage 4 or 5 chronic kidney disease.
9. Any history of or suspected hypersensitivity to the cell product, excipients or possible residual components used in manufacture.
10. Patients who are currently enrolled in another Advanced Therapy Medicinal Product (ATMP) Clinical Trial or Clinical Trial of Investigational Medicinal Product (CTIMP).

*N.B. The Medicines for Human Use (Clinical Trials) Regulations apply in England, Wales, Scotland and Northern Ireland. The regulations allow children aged 16 years and over to give consent to take part in a CTIMP.

### Interventions

The product under investigation is an allogeneic, AAM cell therapy given via peripheral venous infusion at doses from 10^6^ up to 10^9^. The AAM product will be manufactured by the Scottish National Blood Transfusion Service using healthy volunteer donations at a licensed site using methodology described previously, with the addition of an optimised cryopreservation step. This allows for rapid deployment of the AAM product with good macrophage viability.^18 19^ The product has received manufacturing approval from the MHRA. The duration and rates of AAM infusion are given in table 1.

**Table 1:** Duration and rate of intravenous AAM infusion by dose cohort

Dose cohort	Maximum dose (cell number)	Minimum duration (minutes)	Maximum rate (cells per minute)
1	1×10^6^	5	2×10^5^
2	1×10^7^	5	2×10^6^
3	1×10^8^	5	2×10^7^
4	3×10^8^	10	3×10^7^
5	1×10^9^	30	3×10^7^

AAMsalternatively activated macrophages

In addition to AAMs, all patients will be treated with acetylcysteine per standard clinical care. The SNAP regimen for delivering acetylcysteine will be used as recommended by the National Poisons Information Service via TOXBASE (www.toxbase.org) and endorsed as the default standard of care by the Royal College of Emergency Medicine.^20^ The SNAP regimen has a substantially lower rate of adverse drug reactions, which will facilitate the identification of treatment-emergent adverse events due to the cell therapy.

Chlorphenamine 10 mg will be administered intravenously approximately 30 min ahead of macrophage infusion to minimise the likelihood of allergic reaction. If a patient has known allergies or contraindications to chlorphenamine, 10 mg cetirizine hydrochloride will be taken orally instead.

The time interval between overdose and AAM treatment is not defined. Patients in both the progression phase of injury and recovery phase may receive AAMs.

### Outcomes

#### Primary outcome

DLT occurs within 30 days of infusion. DLT is defined as a clinically significant adverse event or abnormal laboratory value assessed as unrelated to disease progression, intercurrent illness or concomitant medications and either meeting the National Cancer Institute Common Terminology Criteria for Adverse Events Grade 3, 4 or 5, or deemed by the independent DMC to be serious enough to prevent an increase in dose of treatment.

#### Secondary outcomes

Within 30 days of dosing:

1. Safety (assessed on days 0, 1, 2, 7 and 30).
  - Adverse events of particular interest (defined as transfusion reaction; macrophage activation syndrome acute respiratory compromise) and all serious adverse events occurring within 30 days of infusion, clinical observations, clinical examination, ECG and safety blood tests.
2. Activity (assessed on days 0, 1, 2, 7 and 30; if available from prior blood serum stored in the biochemistry laboratory, novel liver injury and inflammation markers at additional time points will also be included).
  - Proinflammatory—IL-6, TNF-alpha, IL-12, IL-8 (pg/mL). Anti-inflammatory—IL-10 (pg/mL). Assessed as change from baseline.
  - Liver injury—conventional markers of paracetamol-induced liver injury: ALT (U/L), international normalised ratio, lactate (mmol/L), creatinine (μmol/L). Novel marker of paracetamol-induced liver injury: High mobility group box 1 protein (ng/mL), glutamate dehydrogenase (U/L), cytokeratin-18 (U/L) and miR-122 (copies/mL).^21^ Assessed as change from baseline.
3. Immunogenicity (assessed on day 30).
4. Development of anti-human leucocyte antigen antibodies.

### Recruitment

#### Identification

Potentially eligible patients will be identified by their direct care team at the emergency department, toxicology ward of RIE or from the Scottish Liver Transplantation Unit. An appropriately trained member of the Emergency Medicine Research Group nurses (EMERGE) will assess the patient for study eligibility. Appropriate participant information and informed consent forms will be provided (copies are provided in online supplemental file 3 and online supplemental file 4). Consent will be obtained by an appropriately delegated investigator.

The recruitment period is expected to be 2 years, based on historic attendance numbers of potentially eligible patients.

#### Randomisation

The dose assigned to the sentinel participant in each cohort will be fixed. For the remainder of cohorts 3, 4 and 5 randomisation, using computer-generated pseudo-random numbers produced by the programming team at Edinburgh Clinical Trials Unit (ECTU), will use the allocation ratios across available doses depicted in figure 1A,B as appropriate.

#### Allocation

A random permuted block will be used for each cohort to ensure the required allocation ratio is achieved. Allocation concealment will be maintained by use of a centralised online randomisation system.

#### Blinding

In the randomised dose cohorts, the team that identify potential participants and screen for eligibility (EMERGE) will be blinded to the dose of cells delivered to a patient to minimise selection bias. EMERGE do not deliver any other trial activities after baseline assessments are completed. The investigators may be involved in the consent process and baseline assessments but EMERGE will be solely responsible for identification and screening. Investigators and nurses at the RIECRF, who will be responsible for randomisation, dosing and follow-up, will be aware of the allocated dose, as will the patient. The dose will not be documented in participants’ medical notes to reduce the likelihood of unblinding EMERGE. Blinding can end for individual participants when all participants of a particular dose cohort have been dosed; for instance, the recruiting team no longer needs to be blinded for participants in dose cohort 3 when recruiting to dose cohort 4.

#### Participant retention

If participants are not willing or able to return to the RIECRF, the day 7 and day 30 visits may be performed by a research nurse at the participant’s home. If participants are not willing to have an in-person visit at the RIECRF or at home, follow-up data will be collected by phone and/or using electronic health records. This will be explained to participants during the consenting process. If a participant does not return in person for their day 30 follow-up, this will not constitute a protocol deviation if information regarding clinical events, adverse events and concomitant medications can be collected by phone or from the patient’s medical records for the period between enrolment and the day 30 visit. On enrolment, participants will be provided with contact details for trial staff who can be contacted for advice. In cases of suspected adverse events, an unscheduled visit can be arranged to assess the participant.

### Study assessments

Study assessments are summarised in table 2, with blood panels described in table 3. Research blood samples will be processed and labelled by research staff according to study-specific processing instructions, frozen at −80°C and then transferred periodically to the University of Edinburgh’s Centre for Regenerative Medicine on dry ice for storage in −80°C research freezers until analysis. Any prior blood serum samples received from the central RIE biochemistry laboratory at baseline will be stored and analysed in the same way.

**Table 2:** Study assessments

	Screening	Baseline	Infusion visit	Follow-up 1	Follow-up 2 (if still in hospital)	Follow-up 3	Follow-up 4	Unscheduled visit
	N/A	Day −1 or day 0	Day 0	Day 1	Day 2	Day 7±1 day	Day 30+7 days	
Screening for eligibility	x*							
Consent	x							
Confirmation of eligibility		x						
Medical History		x						
Vital signs		x	x†	x	x	x	x	x
ECG		x	x†‡	x	x	x	x	x
Urine pregnancy test		x§						
Request stored blood samples		x						
Blood panel 1		x						
Randomisation		x¶						
Infusion safety assessments**			x					
Clinical events			x†	x	x	x	x	x
Adverse events			x†	x	x	x	x	x
Concomitant medications		x	x†	x	x	x	x	x
Clinical examination			x†	x	x	x	x	x
Blood panel 2			x‡††	x‡‡	x§§	x		x
Blood panel 3							x	

* The blinded EMERGE team will be solely responsible for the identification and screening of potentially eligible patients.

† Pre-infusion – —observations should be taken a maximum of 60 minutes min prior to commencement of infusion.

‡ 6 hours±30 min postinfusion.In of childbearing potential only.

§ In patients of childbearing potential only.Randomisation will be performed by RIECRF nurses once eligibility has been confirmed. All other screening/baseline assessments will be performed by the blinded EMERGE team.

¶ Randomisation will be performed by RIECRF nurses once eligibility has been confirmed. All other screening/baseline assessments will be performed by the blinded EMERGE team.During infusion: continuous rate and oxygen saturation measurement, with blood pressure, temperature and respiratory rate being measured every minutes (). hours following infusion: measured every minutes (). following infusion: measured every minutes ()

** During infusion: continuous heart rate and oxygen saturation measurement, with blood pressure, temperature and respiratory rate being measured every 5 min (±1 min). 2 hours following infusion: Early Warning Score measured every 15 min (±2 min). 2–6 hours following infusion: Early Warning Score measured every 30 min (±5 min).24±3 hours post start of infusion

†† Pre-infusion if >6 hours since the screening/baseline visit blood draw.

‡‡ 24±3 hours post start of infusion.

§§ 48±3 hours post start of infusion.

EMERGEEmergency Medicine Research Group nursesN/Anot applicableRIECRFRoyal Infirmary of Edinburgh Clinical Research Facility

**Table 3:** Study blood tests

	Blood panel 1	Blood panel 2	Blood panel 3
Blood count	x	x	x
Clotting screen	x	x	x
Urea and electrolytes	x	x	x
Liver function tests	x	x	x
Triglycerides	x	x	x
Ferritin	x	x	x
Paracetamol	x	x	x
Research bloods (secondary outcomes)	x	x	x
C reactive protein	x	x	x
Lactate	x	x	x
Liver antibodies	x		
Immunoglobulins	x		
Alpha fetoprotein	x		
Alpha one antitrypsin	x		
Caeruloplasmin	x		
Calcium	x		
Magnesium	x		
Viral serology	x		
HLA typing	x*		
Anti-HLA antibodies	x†		x

* HLA typing at baseline will be performed in all participants, with the result used to guide selection of donor AAMs.

† In patients with a history of pregnancy or blood product transfusion, this will be a rapid anti-HLA antibody screen. The result will be used to guide selection of donor AAMs.

AAMsalternatively-activated macrophages

### Use of study data

#### Data collection

Study data can be recorded on paper data collection sheets and then entered into the electronic case report form (eCRF) by members of the research team at site. Alternatively, data may be entered directly into the eCRF. All eCRFs will be reviewed and approved by the Sponsor prior to use.

A copy of a blank eCRF can be requested by contacting MAIL.trial@ed.ac.uk.

#### Data management

ECTU will provide and maintain a secure web-based database (Research Electronic Data Capture, Vanderbilt University, USA) compliant with sponsor standard operating procedures. Trained delegated members of the study team will be given password-protected logins to the database for data entry. The data will be stored on a secure server in the University of Edinburgh for the minimum retention period for the study. No directly identifiable information will be entered into the database.

Authorised clients (such as the study or unblinded statistician needing access to produce progress reports to the independent DMC and/or interim or final statistical analyses; the trial manager to produce blinded logistics reports and the quality assurance manager for audit and ongoing quality assurance reports) will be given protected access to relevant data.

#### Data monitoring

A DMC, independent of the sponsor and without competing interests, will be established to oversee the safety of participants in the trial. The terms of reference and composition of the DMC will be detailed in a charter in accordance with sponsor requirements.

The DMC will review safety data and make dose progression recommendations for review by the sponsor and chief investigator. They will also propose changes to the protocol and/or stop the study if indicated.

### Sample size and statistical analysis

If no safety concerns arise which lead to early stopping of the trial or modification of the dose escalation, the design outlined in figure 1A will include 17 participants. If DLTs are observed at the 10^6^ and 10^7^ doses and the trial reverts to a standard 3+3 design, it will include up to 30 participants.

As well as assessment of the primary outcome of safety, this number of participants will enable exploration of any initial signals of efficacy on markers of paracetamol-induced liver injury such as HMGB1, through analysis by isotonic regression modelling. As a guide to the strengths of association detectable in this size of sample, in a one-way analysis of variance with 5 groups and a sample size of 3–4 per group, effect sizes (variance of means divided by within-group SD) between 1 and 2 can be detected with 80% power at a 5% significance level.

A detailed statistical analysis plan has been produced and prospectively documented within the Trial Master File in August 2023. The analysis population for safety outcomes will include all participants receiving any quantity of the investigational allogeneic macrophage cell therapy and will be analysed according to treatment received. Activity outcomes will be analysed for participants receiving any quantity of investigational AAM cell therapy according to intention to treat.

All DLT primary outcome events will be listed by dose and participant alongside their features of seriousness, intensity, relatedness and outcome.

### Safety assessments

Patients will be in the RIECRF for a minimum of 6 hours after cell infusion. There will be continuous heart rate and oxygen saturation measurements during the cell infusion, with blood pressure, temperature and respiratory rate being measured every 5 min (±1 min). The CI or an appropriately trained and delegated subinvestigator, and the RIECRF nurse will be physically present with the patient for the duration of the cell infusion and for 2 hours after dosing with the Early Warning Score (EWS) measured every 15 min (±2 min). From 2 to 6 hours after dosing, the EWS will be measured every 30 min (±5 min), with the RIECRF nurse being physically present with the patient, and the CI or an appropriately trained and delegated subinvestigator being present on site.

Participants will remain in the hospital overnight after dosing. There will be a handover of information from the research team to the clinical team and the principal investigator or an appropriately trained and delegated subinvestigator will be contactable overnight. The patient will be assessed the following day by the research team. Discharge will be a clinical decision of the caring consultant/team.

There are some safety outcomes of particular interest, which have not been reported in previous human trials of macrophage therapy but remain theoretical risks (table 4).

**Table 4:** Safety outcomes of particular interest and the criteria against which they will be assessed

Outcome	Criteria
Acute transfusion reaction	Temperature ≥38°C or raise of 2°C HR >100/min Fall in blood pressure of ≥30 mm Hg Collapse Pain (chest or abdominal or along infusion site) Dyspnoea or oxygen saturation <94% in room air Stridor Mucosal swelling New rash Bleeding diathesis Any other significant changes in clinical condition or observation.
Macrophage activation syndrome	Fever Cytopenia in two or more lineages Hb <100 g/L Platelets <100×10^9^/L Neutrophils <1.0×10^9^/L Hypertriglyceridaemia or hypofibrinogenaemia Triglycerides >2.99 mmol/L Fibrinogen <1.50 g/L Ferritin >500 µg/L
Acute respiratory compromise	SpO2 <92% on air or need for supplemental oxygen Increase in respiratory rate >20/min

HbhaemoglobinHRheart rate

### Monitoring and oversight

All patient enrolment will stop pending advice from the DMC and a representative of the sponsor if one of the following stopping rules have been met:

- A patient dies or requires an escalation in their clinical care with admission to the critical care unit or liver transplant unit after receiving the trial treatment.
- A patient has a safety outcome of particular interest—transfusion reaction, macrophage activation syndrome or acute respiratory compromise as detailed in table 4.
- A suspected unexpected serious adverse reaction occurs.

All clinical events occurring in this study will be assessed against these criteria by an investigator within 24 hours of discovery. If a stopping rule has been met, the investigator will notify the trial team and sponsor within 24 hours of discovery. The trial management team will communicate the recruitment stop to all relevant parties and arrange DMC review.

Following the single infusion of AAMs and beyond the end of the trial, participants will receive standard clinical care. NHS indemnity scheme will apply and the University has No Fault Compensation Insurance in place.

### Study amendments and current status

At the time of submission, the protocol in operation is V.5.0, and this is reflected in this manuscript. Protocol changes and implementation dates are provided in online supplemental file 1. Recruitment started on September 2023. The trial is following scenario 1 and the study team has recruited the first participant in cohort 3. We are on target to complete recruitment by the end of 2025.

Participants 1 and 2 were recruited under protocol V.3.0. Participant 3 was recruited under protocol V.5.0. The protocol changes have served only to clarify processes and would not have impacted participant eligibility.

Any future amendments to the protocol will be communicated to all relevant parties, including trial registries and publishing journals.

## Ethics and dissemination

The trial will be conducted according to the ethical principles of the Declaration of Helsinki 2013 and has been approved by North East—York Research Ethics Committee (reference 23/NE/0019), NHS Lothian Research and Development department and the MHRA. Good Clinical Practice regulations will be followed and written informed consent will be obtained from all participants.

No trial results will be shared while the trial is ongoing. When the trial completes, data will be shared via presentation, publication and may be available on request from the trial chief investigator, decided on a case-by-case basis. Data sharing may be restricted in line with patent or commercial requirements for as short a time as possible.

Results will be disseminated by peer-reviewed publication, conferences and linked on isrctn.com. Publication content will follow the Consolidated Standards of Reporting Trials-DEFINE reporting guidance extension for dose-finding studies.^22^ Ownership of data arising from this study resides with the study team and their respective employers. The study team will follow the International Committee of Medical Journal Editors guidelines on authorship. Requests for data access should be sent to the corresponding author.

## supplementary material

10.1136/bmjopen-2024-089417 online supplemental file 1

10.1136/bmjopen-2024-089417 online supplemental file 2

10.1136/bmjopen-2024-089417 online supplemental file 3

10.1136/bmjopen-2024-089417 online supplemental file 4
