# Supplementary material for: Macrophage Therapy for Acute Liver Injury (MAIL): a study protocol for a phase 1 randomised, open-label, dose-escalation study to evaluate safety, tolerability and activity of allogeneic alternatively activated macrophages in patients with paracetamol-induced acute liver injury in the UK
Source: BMJ Open. 2024 Dec 9;14(12):e089417. doi: 10.1136/bmjopen-2024-089417 (PMC11628987; doi:10.1136/bmjopen-2024-089417)
Supplement: Supplementary file 5 [file bmjopen-2024-089417.reviewer_comments.pdf]

PEER REVIEW HISTORY

BMJ Open publishes all reviews undertaken for accepted manuscripts. Reviewers are asked to complete a checklist review form and are provided with free text boxes to elaborate on their assessment. These free text comments are reproduced below.

ARTICLE DETAILS

Title (Provisional)

Macrophage Therapy for Acute Liver Injury (MAIL): a study protocol for a Phase 1 Randomised, Open-Label, Dose-Escalation Study to Evaluate Safety, Tolerability, and Activity of Allogeneic Alternatively Activated Macrophages in Patients with Paracetamol-induced Acute Liver Injury in the United Kingdom

Authors

Humphries, Christopher; Addison, Melisande; Aithal, Guruprasad Padur; Boyd, Julia; Briody, Lesley; Campbell, John DM; Candela, Maria Elena; Clarke, Ellise; Coulson, James; Downing-James, Nicholas; Fontana, Robert J.; Geddes, Ailsa; Grahamslaw, Julia; Grant, Alison; Heye, Anna; Hutchinson, James A; Jones, Ashley P.; Mitchell, Fiona; Moore, Joanna; Riddell, Alice; Rodriguez, Aryelly; Thomas, Angela; Tucker, Garry; Walker, Kim; Weir, Christopher; Woods, Rachel; Zahra, Sharon; Forbes, Stuart J; Dear, James W

VERSION 1 - REVIEW

**Reviewer**	**1**
**Name**	**Jaeschke, Hartmut**
**Affiliation**	**The University of Kansas, Pharmacology, Toxicology & Therapeutics**
**Date**	**15-Jul-2024**
**COI**	**None**

---

1. It is unclear from the protocol if the investigators aim to consistently treat the patients in terms of the time interval between the overdose and the AAM treatment. They state that patients should have at least >1,000 U/L serum ALT activities to be eligible for the study. However, it is unclear if they plan to treat patients during the progression phase of the injury or wait until ALT values plateau and then decrease (recovery phase). Because the future objective of this treatment is to support recovery, it may be useful to only focus on a later treatment (after the injury phase) for the safety assessment. The investigators should discuss their treatment timing.

2. The investigators will use allogeneic alternatively activated macrophages (AAM) manufactured as described in ref. 16. Are the generated AAMs stored frozen and thawed before use or are only freshly prepared AAMs being used? Ref. 16 describes loss of cell viability and other adverse effects when macrophages were frozen and thawed. This issue needs to be discussed.

**Reviewer**	**2**
**Name**	**Copple, B**
**Affiliation**	**Michigan State University**
**Date**	**05-Aug-2024**
**COI**	**None**

This phase I clinical study will determine the safety of allogeneic macrophage therapy for the treatment of paracetamol-induced acute liver injury. The rationale for this study stems from findings in mice which demonstrated a modest improvement in measures of liver injury and cell proliferation after infusion of paracetamol treated mice with alternatively-activated macrophages (AAMs). Overall this is a well-designed study to investigate the safety of allogeneic macrophage therapy to alleviate paracetamol-induced liver injury in overdose patients. Although I have no suggestions to improve the protocol, I do have a couple of comments for the authors to consider.

1) The protocol will utilize allogeneic macrophages because these patients often present with monocytopenia limiting the number of monocytes available for differentiation and the monocytes would most likely be exposed to a number of different cytokines and immunomodulatory factors that could impact their differentiation. The authors should consider, however, that the allogeneic macrophages will be entering this same cytokine-rich environment which could impact the ability of these cells to facilitate liver repair. For instance, it was recently demonstrated in mice, with clinically relevant paracetamol-induced acute liver failure, that monocytes accumulate in the perinecrotic regions but fail to enter the lesions and clear dead cell debris. Paradoxically, this was corrected in part by reducing levels of interleukin-10. Because paracetamol overdose patients with the worst outcome present with the highest levels of interleukin-10 (compensatory anti-inflammatory response syndrome; CARS), it is possible that this could similarly limit the efficacy of infused macrophages in patients with the most severe acute liver failure.

2) Studies have shown that macrophages clear ALT and potentially other proteins from the blood after liver injury. As a result, increasing the circulating pool of phagocytic macrophages in patients with acute liver injury may reduce ALT and other endpoints of liver injury, such as

AST and HMGB1, through increased clearance from the blood rather than a reduction in liver injury.

VERSION 1 - AUTHOR RESPONSE

Reviewer: 1
Prof. Hartmut Jaeschke, The University of Kansas
Comments to the Author:

1. It is unclear from the protocol if the investigators aim to consistently treat the patients in terms of the time interval between the overdose and the AAM treatment. They state that patients should have at least >1,000 U/L serum ALT activities to be eligible for the study. However, it is unclear if they plan to treat patients during the progression phase of the injury or wait until ALT values plateau and then decrease (recovery phase). Because the future objective of this treatment is to support recovery, it may be useful to only focus on a later treatment (after the injury phase) for the safety assessment. The investigators should discuss their treatment timing.
Thank you. As this is a phase 1 trial and does not primarily seek to identify product efficacy, the time interval between overdose and AAM treatment is not defined. A clarification has been added to 'Interventions'.
2. The investigators will use allogeneic alternatively activated macrophages (AAM) manufactured as described in ref. 16. Are the generated AAMs stored frozen and thawed before use or are only freshly prepared AAMs being used? Ref. 16 describes loss of cell viability and other adverse effects when macrophages were frozen and thawed. This issue needs to be discussed.
Thank you. We have added further text and another reference to clarify that this is delivered as a cryopreserved product, which has been developed since the referenced publication to deliver thawed macrophages of sufficient quality.

Reviewer: 2
Dr. B Copple, Michigan State University
Comments to the Author:

This phase I clinical study will determine the safety of allogeneic macrophage therapy for the treatment of paracetamol-induced acute liver injury. The rationale for this study stems from findings in mice which demonstrated a modest improvement in measures of liver injury and cell proliferation after infusion of paracetamol treated mice with alternatively-activated macrophages (AAMs). Overall this is a well-designed study to investigate the safety of allogeneic macrophage therapy to alleviate paracetamol-induced liver injury in overdose patients. Although I have no suggestions to improve the protocol, I do have a couple of comments for the authors to consider.

1. The protocol will utilize allogeneic macrophages because these patients often present with monocytopenia limiting the number of monocytes available for differentiation and the monocytes would most likely be exposed to a number of different cytokines and immunomodulatory factors that could impact their differentiation. The authors should consider, however, that the allogeneic macrophages will be entering this same cytokine-rich environment which could impact the ability of these cells to facilitate liver repair. For instance, it was recently demonstrated in mice, with clinically relevant paracetamol-induced

acute liver failure, that monocytes accumulate in the perinecrotic regions but fail to enter the lesions and clear dead cell debris. Paradoxically, this was corrected in part by reducing levels of interleukin-10. Because paracetamol overdose patients with the worst outcome present with the highest levels of interleukin-10 (compensatory anti-inflammatory response syndrome; CARS), it is possible that this could similarly limit the efficacy of infused macrophages in patients with the most severe acute liver failure.
Thank you, we will bear this in mind when interpreting our study findings.

2. Studies have shown that macrophages clear ALT and potentially other proteins from the blood after liver injury. As a result, increasing the circulating pool of phagocytic macrophages in patients with acute liver injury may reduce ALT and other endpoints of liver injury, such as AST and HMGB1, through increased clearance from the blood rather than a reduction in liver injury.
Thank you. We recognise that in the absence of biopsy, our biomarkers of interest represent proxy markers, with limitations. We are measuring a range of biomarkers including RNA species (miR-122) which may facilitate inferences about the liver specificity of an effect on ALT. A phase 2 trial would seek to explore this issue further.

VERSION 2 - REVIEW

**Reviewer**	**1**
**Name**	**Jaeschke, Hartmut**
**Affiliation**	**The University of Kansas, Pharmacology, Toxicology & Therapeutics**
**Date**	**28-Oct-2024**
**COI**	

The authors satisfactorily addressed the comments

**Reviewer**	**2**
**Name**	**Copple, B**
**Affiliation**	**Michigan State University**
**Date**	**22-Oct-2024**
**COI**	

Good luck with the study.
